# The influenza of 1918: Evolutionary perspectives in a historical context

**DOI:** 10.1093/emph/eoy024

**Published:** 2018-08-31

**Authors:** Margaret Humphreys

**Affiliations:** Department of History, Duke University, Durham, NC

**Keywords:** influenza, World War 1, virulence, social determinants of health

## Abstract

The 1918 influenza pandemic was the deadliest in known human history. It spread globally to the most isolated of human communities, causing clinical disease in a third of the world’s population, and infecting nearly every human alive at the time. Determination of mortality numbers is complicated by weak contemporary surveillance in the developing world, but recent estimates put the death toll at 50 million or even higher. This outbreak is of great interest to modern day epidemiologists, virologists, global health researchers and evolutionary biologists. They ask: Where did it come from? And if it happened once, could it happen again? Understanding how such a virulent epidemic emerged and spread offers hope for prevention and strategies of response. This review uses historical methodology and evolutionary perspectives to revisit the 1918 outbreak. Using the American military experience as a case study, it investigates the emergence of virulence in 1918 by focusing on key susceptibility factors that favored both the influenza virus and the subsequent pneumococcal invasion that took so many lives. This article explores the history of the epidemic and contemporary measures against it, surveys modern research on the virus, and considers what aspects of 1918 human and animal ecology most contributed to the emergence of this pandemic.

The 1918 influenza pandemic was the deadliest in known human history. It spread globally to the most isolated of human communities, causing clinical disease in a third of the world’s population and infecting nearly every human alive at the time. Determination of mortality numbers is complicated by weak contemporary surveillance in the developing world, but recent estimates put the death toll at 50 million or even higher [1–8]. This outbreak is of great interest to modern day epidemiologists, virologists, global health researchers and evolutionary biologists. They ask: Where did it come from? And if it happened once, could it happen again? Understanding how such a virulent epidemic emerged offers hope for prevention and strategies of response.

As a historian of science with a focus on public health, I aim to set the stage for later articles in this collection using historical methodology. As a physician with interests in evolutionary medicine, I weave evolutionary perspectives into this historical context, focusing on the American military experience as a case study to investigate the emergence of virulence in 1918 by focusing on key susceptibility factors that favored both the influenza virus and the subsequent pneumococcal invasion that took so many lives. I structure this article around the history of the epidemic and contemporary measures against it, survey modern research on the virus, and consider what aspects of 1918 human and animal ecology most contributed to the emergence of this pandemic.

## HISTORY OF THE PANDEMIC (I)

It is important to realize that there were in fact two linked epidemics—influenza and subsequent bacterial pneumonia—which together generated high case mortality rates. Characteristics of the influenza strain determined its contagiousness, its widespread diffusion and its virulence. Bacterial pneumonias occur in epidemic form rarely, but it was widely noted by contemporaries (and later researchers) that the patients who died of ‘influenza’ actually died of a secondary bacterial pneumonia. There is no suggestion that this was a particularly virulent strain of pneumonia; rather it opportunistically invaded vulnerable influenza patients. This article will explore reasons why some patients, particularly those from rural areas, may have been more susceptible to death from this secondary bacterial infection. One set of factors determined whether a person would become severely ill with influenza; severe infection with bacterial pneumonia may have had other determinants.

The influenza epidemic occurred in at least three waves, as visualized in Europe and America. The first wave appeared in the spring of 1918, in a well-documented outbreak at a military base in the farm state of Kansas. From there it spread with American troops throughout the nation and overseas on crowded trains and troop ships. It moved quickly through the congested military camps of Europe and on to east and south Asia, infecting all sides of the conflicts without regard for nationality. This first wave was a minor outbreak, but not a major killer. Those who acquired it were lucky, however—it apparently offered some immunizing protection for the virulent variants of the pathogen to come [9].

That second wave appeared at several locations spontaneously at the end of August 1918. Thousands of miles apart, men in Freetown, Sierra Leone; Brest, France; and Boston, Massachusetts fell ill with a fever that ended in death for many. One physician in Boston, aghast at the previously healthy soldiers dying all around him, predicted that the disease would spread quickly over the country, infecting 30–40% of the population, and killing 1 in 20 of those infected [5]. As men in the armed forces moved from port to port, and camp to camp for training of various sorts, the disease followed them. By Easter 1919, some 550 000 Americans had died of the disease, with more than 11 000 dead in Philadelphia just in the month of October. Some thought it was the end of the world. One estimate echoed the Boston prediction, putting the apparent infection rate at a quarter of the population—25 million or more [5].

One characteristic of the epidemic particularly puzzled observers. Unlike usual influenza, which was most lethal among infants and the aged, this outbreak also targeted young adults aged 20–40. Explaining such a ‘W’ mortality curve was a major challenge both for contemporaries and modern analysts. Victor Vaughan, one America’s leading public health experts, saw it as a reflection of the high impact of the epidemic on men in the military, who were, after all, mostly young adults [10]. Modern analysts invoke other possibilities, as will be discussed below [11]. Later in this volume David Fedson considers age-related influenza mortality rates in detail, with evolutionary implications and suggestions for public health response [12].

The other striking novelty in this epidemic’s statistics was its high case mortality rate, which reached 5% or higher in some populations. Usual epidemics of influenza in the era prior to modern medicine killed no more than one patient in a thousand [5]. This epidemic was something new under the sun, almost certainly the result of viral mutation. It was a true pandemic, i.e. the appearance of a novel organism for which many had no prior immune experience.

## MODERN RESEARCH ON THE 1918 VIRUS: UNDERSTANDING THE EMERGENCE OF NOVELTY AND VIRULENCE

Influenza is a blanket term for a family of respiratory infections caused by single-stranded, negative sense RNA viruses which occur in four types, labeled A, B, C and D. Influenza A causes the majority of severe disease in humans. Its genome is made up of eight segments which in turn code for the hemagglutinin (HA) and neuraminidase (NA) surface proteins, as well as other viral components. The HA and NA proteins act as the principal antigens against which the body mounts an immune response. As Nelson and Holmes note, ‘Severe influenza pandemics can occur following a sudden antigenic shift—when a reassortment event generates a novel combination of HA and NA antigens to which the population is immunologically naïve’. [13]

How does this reassortment event happen? It has become clearer over the past two decades that human influenza exists on the periphery of a vast global network of avian influenzas. All 16 HA subtypes and 9 NA subtypes that have been identified are found within this ecosystem. They live in wild birds, including ducks, geese, swans and various shore birds. And those birds often migrate, moving in the northern hemisphere from the arctic to the tropics and back again. From these birds, via fecal-oral spread in water and infected soils, the virus can move to domesticated birds as well as mammals, including bats, horses, pigs and humans [14]. While evolution of the virus includes antigenic shift and response to positive evolutionary pressures, the possibility that a single host may harbor multiple viral strains ‘facilitates reassortment between isolates that co-infect the same host cell’.[13] As birds gather and interact, they exchange the avian influenza viruses promiscuously.

This process can connect Asia to North America. How were these areas linked? One known method is the transport of live swine globally, a trade that facilitated the emergence of the H1N1 outbreak in 2009 [15, 16]. Another is the flight of wild birds. It has recently been demonstrated that an avian flu discovered in Korea traveled via wild waterfowl into the United States. Asian waterfowl fly north to summer in the arctic and mix with North American birds. These migration patterns create a broad passageway that connects east Asian avian viruses to North America [17]. The transition of novel influenza antigens to the United states did not require the movement of humans or other mammals from Asia or elsewhere. There was a children’s ditty popular in 1918. ‘I had a little bird, its name was Enza. I opened the window and in flew Enza’. Exactly? [5]

In a series of papers published in the late 1990s, Jeffery Taubenberger, Ann Reid and colleagues described the genome of the 1918 influenza virus. They had found it in dusty pathological specimens from the U.S. Armed Forces Institute of Pathology and through *in situ* biopsy of a victim buried in permafrost at Brevig Mission in Alaska [18–21]. In the two decades since discovery scientists have probed these genomes for clues about the origin of the virus and its unusual virulence.

With the reconstituted 1918 viral genome in hand, Gavin Smith *et al.* argued in 2009 that molecular clock phylogenetic methods suggested ‘over a number of years, avian gene viral segments have entered mammalian populations where the viruses may have undergone reassortment with the prevailing human virus’. The zoonotic sources for the 1918 influenza virus ‘remain ambiguous’, but they believed that ‘given the frequent interspecies transmission of influenza viruses between swine and humans, it is most likely that such reassortment events occurred in swine before pandemic emergence’. [22]

Recent work by Worobey, Han and Rambaut challenges this notion, using molecular clock methodology that takes into account host-specific evolutionary rates. They argue instead that the novel H1N1 influenza virus of 1918 emerged via reassortment between an older H1 human influenza A lineage and an avian virus. They find that the 1918 human virus then moved into pigs, creating a swine flu epidemic noted at the time. This in turn explains the finding that swine flu specimens from the 1930s match the now identified 1918 human virus closely. In other words, the 1918 virus was a mix of human and avian, with no swine donation to its genome [23]. Oliver Pybus of Oxford, was not entirely convinced. About the paper he said, ‘It shows the evidence for a pig origin is a lot weaker, but it’s almost impossible to completely shut the door on that’. [24] For the moment, however, the issue appears to be weighted in favor of human + avian. This question of avian + swine > human as opposed to avian > human > swine may be important for estimating the danger and locales of future epidemics. It also lets us at least speculate about why the first American influenza outbreak occurred where it did, in rural Kansas.

A second important point emphasized in Worobey, Han and Rambaut is their explanation of the W curve, the high mortality of young adults. They draw on the concept of ‘HA imprinting’, the idea that are two subgroups of the HA antigen. Persons will have lifelong immunological memory of the subgroup of HA antigens that was produced during the first influenza episode of their life. This in turn generates growing herd immunity against animal infections (avian, swine) from that group. The age curve of the 1918 influenza suggests that those born before the great influenza epidemic of 1889 were exposed to the same HA group of the 1918 flu, and therefore had some immunity to it. But the young men and women born later, the young adults so affected in 1918, had only met the other HA subgroup—in this case H3N8 and hence their immune systems were less effective against the 1918 outbreak. Worobey, Han and Rambaut conclude, ‘We hypothesize that childhood exposure to an H3N8 virus may have made some young adults in 1918 a sort of temporal counterpart to highly vulnerable geographical isolated populations, inducing suboptimal immunity that tilted the odds in favor of secondary infection with the wide range of bacterial pathogens that cause most influenza-related mortality’. [As we shall see later, actual geographical isolation (prior to wartime conditions) may have also played a role [11, 23].

An alternate explanation posits that the immune reaction, far from being too weak in young adults, was overly strong. This argument builds on observations of avian influenza viruses that have crossed the species barrier to kill humans. As Liu *et al.* [25] note, ‘These infections in humans are accompanied by an aggressive pro-inflammatory response and insufficient control of an anti-inflammatory response’. In the lungs this can result in swollen alveoli full of debris and proteinaceous fluid. Rapid cyanosis, the blue skin color that indicates inadequate oxygenation would result—and was indeed a symptom of severe 1918 cases. In that immune function fades with age, it follows that young victims would be most vulnerable to this hyper-response.

It is possible that some men died quickly from such a cytokine storm phenomenon, but recent research supports death, instead, from a secondary pneumonia. Morens, Taubenberger and Fauci surveyed stored pathology slides of lung tissue from 1918 soldiers who died, as well as reviewed contemporary literature that reported on victim autopsies. They concluded that a two-step process explained mortality. First, ‘the virus, highly cytopathic to bronchia and bronchiolar epithelial cells, extends rapidly and diffusely down the respiratory tree, damages the epithelium sufficiently to break the mucociliary barrier to bacterial spread’. Such action created a direct pathway for bacterial invasion, and an environment, characterized by cell necrosis and proteinaceous fluid, optimal for bacterial growth. From bacterial studies done at the time they concluded that almost half of the cases grew pneumococcus (*Streptococcus pneumoniae*) or a mixed bacterial infection that included pneumococcus. *Streptococcus pyogenes* and *Staphylococcus aureus* were also frequently found. Massive bacterial pneumonia killed these patients. The exact relationship between the virus and the bacterium remains, they report, poorly understood [26].

As noted, the second wave of this influenza was undeniably characterized by high virulence. Virulence can be defined starkly as the likelihood of host death, or by using various measures of host damage, such as weight loss. Given the high mortality from the 1918 pandemic, it is seems likely that some sort of mutation led to increased virulence in the influenza virus. Or, it is possible, the key mutation was greater infectivity. The mortality rate stayed at about 5%, but many, many more people contracted the virus. Success, in viral terms, involves both rapid reproduction in the infected host and effective transmission to susceptible host [27]. Yet rapid reproduction, which involves evasion or alteration of host defenses, destruction of host tissues and consumption of host resources, may diminish transmission by immobilizing or killing the host, limiting spread to only its immediate environs. This polarity, described in the ‘trade-off hypothesis’, predicts that less virulent infections will evolve in settings where likelihood of transmission is low, so that the ambulatory host can move and spread the infections to others. And the contrary may be the case—highly virulent, even deadly microorganisms can successfully spread if patient ambulation is not a factor. Thus in vector-borne diseases or those spread through fecal contamination of the water supply, the organism can escape from the sedentary patient. But for respiratory spread, the next host may need to be found within a few feet of the coughing source [28, 29].

Paul Ewald relied heavily on the trade-off hypothesis in his 1994 book on the evolution of infectious disease. He particularly emphasized the likelihood of influenza transmission in the crowded trenches of World War I, and the ways in which ambulance transfer to hospitals helped spread the virulent strain [28]. Since the initial presentation of the trade-off hypothesis (which replaced an older theory that all microorganisms evolve toward lower virulence), it has been much disputed. While it makes sense at the macro-level, it has been quite difficult to prove experimentally, or from statistical studies of natural systems. As Alizon *et al.* [30] note, ‘The appeal of the trade-off hypothesis results from its simplicity and generality’, but ‘showing evidence of a trade-off empirically is highly complicated’.

**Figure 1.**
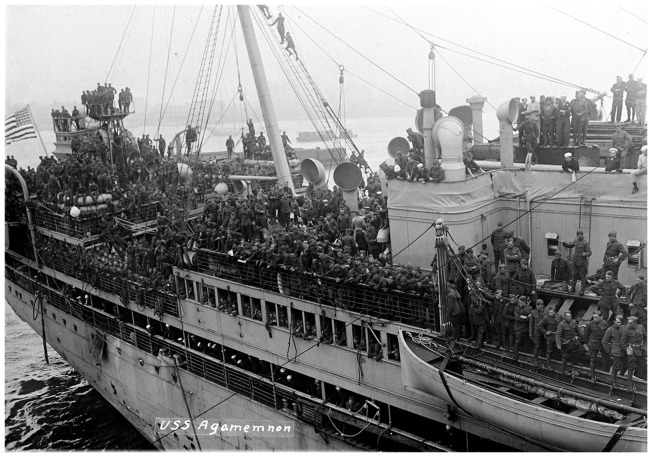
Homeward-bound troops crowd the Agamemnon’s deck in 1919, arriving at New York Harbor. ID # 3004, U.S. Naval History and Heritage Archive

One refinement of the theory seeks to incorporate both population structure and prior viral-induced immunity levels into epidemic models. The stable conditions that exist when a population is relatively closed can be changed dramatically if it is exposed to new pathogens and/or suddenly in communication with host populations of differential susceptibility. For example, Boots, Hudson and Sasaki found that a virulent rabbit hemorrhagic virus emerged when a large cohort of rabbits was transported by plane from Germany to China in 1984. Had the mutated virus appeared only in Germany, it would have found that most proximal rabbits had some form of immunity to the older virus from which it had recently evolved. But as it was carried by air travel to susceptible populations, the virulent strain was favored and rapidly created a new evolutionary stable state [31, 32]. It is certainly true that World War I disrupted many stable local human populations, prompting an unusually high degree of mixing not only due to troop movements, but also to the sort of refugee migrations that accompany any major conflict [Fig. 1].

A second factor in transmission is the number of susceptible hosts in the population. In the modern era, the number of people who have been vaccinated against a virus, or who have survived prior infection with the virus will affect successful spread. For some viruses, experiencing a case of the disease or acquisition of vaccine can immunize for years, or even a life time. But influenza mutates rapidly and often, so vaccination must be repeated yearly to match the new strains. Still, prior exposure in childhood to influenza disease can affect lifetime susceptibility.

Likelihood of prior infection is directly tied to age; the older the potential host the more viral experience the body has. Countering this advantage is immune senescence, the fading of immune function with age. Acquired immunity is also determined by the density of population in the areas where the individual lived. Someone raised in rural farm country may have never encountered a virus common in cities. This phenomenon was evident in the American Revolutionary and Civil Wars. George Washington’s American volunteers were far more likely to fall ill of smallpox than the British troops who had been recruited from the slums of England’s cities, where they had either acquired infection naturally or been inoculated on enlistment [33, 34]. In the American Civil War one historian has estimated that it took about a year for men from rural New York State to become ‘seasoned’, that is, to run through the various ‘childhood’ diseases (such as measles, mumps, chickenpox) prevalent in camp and acquire the immunological experience that urban troops had already acquired from mild childhood infection [35].

Virulence is also directly affected by the general strength of the host’s immune system, which in turn can be depressed by malnutrition, fatigue and high levels of stress that can mimic the impact of oral corticosteroids. Measles, for example, is much more virulent in the setting of protein deficiency. The 10–50% lethality of measles in tropical Africa and Australian aborigines offers evidence that malnutrition multiplies death rates from this disease by a magnitude of 300-fold [27]. The varied populations distressed and displaced by war may have suffered under many immunological insults.

Modern medical ideas about prior immunological exposures and the varied aspects of the social determinate of health let us consider again the contemporary reports on the first major outbreak of influenza in the United States. The specific research on the 1918 virus, particularly the focus on secondary pneumonia, will be supported by the physicians writing at the time who tried to understand the outbreak. And a review of public health response to the epidemic will likewise be clearer in light of such research.

## INFLUENZA IN THE AMERICAN HEARTLAND: ECOLOGY AND POPULATIONS

The first well-documented outbreak of influenza in 1918 occurred at Camp Funston, Fort Riley, Kansas. A variety of authors have argued for an earlier appearance—in France, in China, in a small town in west Kansas. Worobey, Cox and Gill (in review) review these hypotheses later in the this issue [36–38]. Such records may describe the earlier influenza viruses which modern day genomic studies suggest were circulating in preceding years. Be this as it may, the Kansas outbreak was studied in detail at the time and provides an unambiguous case study of ‘first wave’ influenza in 1918 America [39]. It also can stand as an exemplar of how amenable the army camp environment was for the emergence of virulence.

If the human influenza virus can undergo reassortment through contact with avian or swine influenza viruses, were there domestic birds and pigs in the vicinity of Camp Funston? The same railroad that brought men to the camp passed through Kansas City, home to the largest feed lot in the state with room for 10 000 hogs. The Kansas City slaughter house could process 140 000 hogs a day [40]. The counties that encompassed Fort Riley and Camp Funston had a census of 34 000 hogs in 1917. Poultry were not counted in such detail, but Kansas did produce 14.8 million dollars worth of poultry meat and eggs in 1918 [41]. In the absence of refrigeration, live chickens and hogs would have arrived to feed the men at Camp Funston and been kept there on the camp farm until slaughtered. On the importance of human–avian and human–swine interactions in the transmission of influenza viruses, see the article by Bailey *et al.* later in this issue [42].

**Figure 2.**
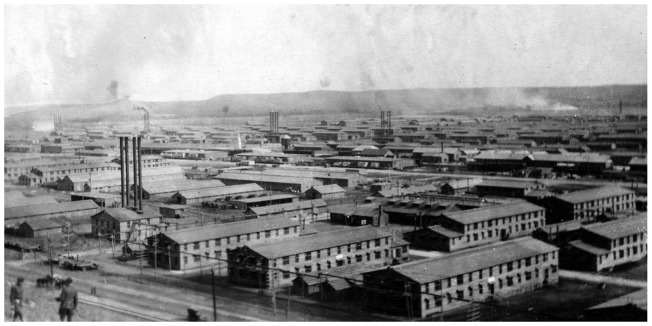
Post card of Camp Funston, 1918. In the author’s possession

Camp Funston was the largest US Army training camp built stateside during World War I [Fig. 2] More than 40 000 troops could be housed there at one time, in around 3000 buildings. The sleeping barracks measured 43 by 140 ft, with two stories to each building. There were 150 beds on each floor, in one large open sleeping room. The camp also had theaters, social centers, infirmaries, schools, animal pens and workshops. The men jammed into the theatre to hear frequent entertainments, including an 80-piece orchestra from St. Louis [43].

All of these camp arrangements were implicated in late 1918 by Vaughan and Palmer who highlighted the assembly rooms as key to the transmission of air-borne organisms in camp life. ‘The acute respiratory diseases are transmitted by the transference of organism from the respiratory tract of one man to those of another. To put it bluntly, ‘by spitting into one another’s face’.’ For example, ‘In an assembly hall in one of our camps where several thousand men are seated night after night, if every man sits upright and moves his head neither backward nor forward, the greatest distance between his nose and that of the man in front or behind is 26 inches, and to the right or left, 16 inches’. Since, they added, ‘in such an assembly with one-half of the men coughing, one can have some idea of the extent to which respiratory bacteria are being transmitted’. The men did a lot of regular spitting as well, coating the sidewalks and floors with bubbly sputum [10].

Adding to such opportunities for aerial spread in close quarters was the path from mouth to hand to mouth. This might be amplified by the common practice for washing mess kits. As described in one account, ‘The soldiers pass in line past the pail of wash water, each dipping his dishes in the water and using his hand as a mop in cleansing. Units of as many as 200 men have used the same wash water without changing’. Such water was tepid, offering no threat to microorganisms. ‘As each man contaminates the wash water with his own variety of bacteria’, he at the same time acquired the prior bacterial donations of others. Such contacts could also occur in bathrooms with communal towels, or water buckets with a shared cup. The modern rapid transmission of norovirus from unwashed hands to surfaces to new hands to mouths illustrates how frequently humans bring fingers to their faces [9].

The trains that brought men to Camp Funston, or transported them eastward to ports of departure for Europe were likewise crowded. The United States entered the war with such inadequate train stock that the government soon nationalized the railroads in order to move military supplies and personnel. Between January and November 1918, those trains carried 6.5 million men, with a maximum in July 1918 of more than a million. Once the United States entered the war in April 1917 the rapid mobilization of four million men required speed and consequent overcrowding of camps, trains, and troop ships [44].

When the influenza first appeared at Camp Funston in March 1918, the epidemic blossomed quickly. Eugene L Opie, who authored the landmark study of this event, was a lieutenant colonel in the medical corps who had trained under William Welch at Johns Hopkins, done research at the Rockefeller Institute, and been dean (and chair of pathology) at the Washington University Medical School in St. Louis prior to enlisting in the army [45]. The original paper on influenza at Camp Funston had as its main subject the appearance of pneumonia in the camp from its founding in August 1917 to the end of August 1918. Opie was particularly interested in the racial diversity of pneumonia cases over the summer: ‘Pneumonia of this period has in considerable part affected newly drafted negro troops from Southern states, namely Louisiana and Mississippi’. Among the 5982 black men arriving over four days in late June, there were 69 cases of pneumonia, whereas among the 12 000 white draftees who arrived in June, only one case of pneumonia occurred. A further cohort of 5997 black men arriving in July developed 20 cases of pneumonia while there were only eight cases among the 15 000 white recruits in that month [39].

Opie investigated factors that might have distinguished the treatment of these men. First he explored ‘conditions that depress resistance, such as exposure to cold or wet, exposure to dust, fatigue’ and exposure to the typhoid vaccine. ‘We have obtained no evidence that these negro drafts which [*sic*] have developed pneumonia have been exposed to cold or wet during transportation to the camp, or after their arrival’. The men had not been exposed to the dust storms that apparently plagued the area in 1918 to any greater degree than white troops. And their work load was similar; they ‘have not been required to perform prolonged or severe drill; their work has not been heavier than that of white troops’. While Opie might have missed subtle differences in black troop care—delay in being allowed to go to sick call, or less nutritious food, or different clothing—still, it seems he did inquire about social determinants of health, and did not find an explanation [39]. He also did not consider pre-existing malnutrition or other conditions, such as anemia from hookworm, a common infestation in the early twentieth century south [46].

Instead, Opie found the answer came from culturing the mouths of healthy men, white and black, and comparing the results to the organisms present in infected sputum. Black men from Louisiana and Mississippi were sick with varieties of pneumococcus commonly present in the mouths of healthy white men. In other words, the black troops from Louisiana and Mississippi were exposed for the first time to these varieties of pneumococcus, whereas the healthy men, especially the white troops, had met it before and developed some immunity. Opie concluded the difference was by region of origin, not race. There were no white men at Camp Funston from Louisiana or Mississippi for comparison [40]. Southern states had objected to the amassing of large number of negro troops in the south, so they were often transported to camps beyond its borders [47]. This study supported the argument that immunological naiveté was one factor in the dissemination of pneumonia.

Opie’s discussion of the influenza epidemic appears almost by accident, and in conjunction with his argument that pneumonia outbreaks followed influenza by a fairly regular pattern of 5 days lag from first influenza case. There had been influenza/pneumonia over the winter, which he said resembled the influenza and pneumonia of civilian life. Whether these infections were similar to the severe influenza noted in Haskell, Kansas in February 1918 is unknown [37, 48]. Did the ‘usual’ influenza mutate, or did a new virus arrive? Unknown. But clearly something dramatic happened in early March. Opie described symptoms that did not exactly match ‘usual’ influenza, but said that the epidemiological pattern seemed to confirm its presence.

The symptoms were non-specific. The fever was unimpressive, ranging from 99 to 103°F. ‘The patient was prostrated, had severe headache, and complained of aching pains in the muscles of the back of the neck, of the lumbar region and at times of the arms and legs’. Some were sent to the meningitis ward due to the neck pain and fever. Runny nose and cough were not common, and bronchitis was rare. A few had abdominal pain and were sent for observation to the surgical ward. In summary, ‘it was evident that there were no characteristic physical signs associated with the disease’. Its course spanned 24–48 h when the fever dropped and the patient recovered. Between March 4 and March 29 some 1127 men were sent to base hospital with these symptoms, with more cared for in the infirmaries near their barracks. This amounted to almost 4% of the men on the base. Most of the men affected had been at the camp 3–6 months. After the first wave, new rounds of the disease began when large numbers of draftees arrived at the camp. He reported no mortality from the influenza per se, but 14–20% from the following pneumonia [39].

Opie’s paper describes the pattern of later severe influenza in miniature. Death followed appearance of pneumococcal pneumonia but only a quarter or so of the 1730 influenza cases progressed to pneumonia, and 18% of that subset died. There were 429 cases of pneumonia following the influenza outbreak and 77 deaths. If we consider the pneumonia to be a later manifestation of influenza, then influenza caused a 5% case mortality rate. Another 390 men developed ‘acute bronchitis’, presumably a milder version of the same phenomenon. So the 1918 influenza was showing its true colors, only it was a pale wash not a vibrant slash. It affected young adults, it facilitated secondary bacterial infections of the respiratory tree, and it increased mortality. No one paid much attention, and apparently no one preserved any sort of lung tissue that would enable modern virologists to code its RNA and compare it with the lethal strain that emerged in late August 1918 [39].

In a summary essay published in 1921, Opie drew conclusions that are concordant with modern assumptions about the epidemic. He recognized that the influenza virus destroyed the local immune protection in the bronchial tree. ‘Microscopic study demonstrates that the changes in the bronchial walls are such as to destroy the defences [*sic*] against invasion by microorganisms’. If followed by exposure to pneumococci, lobar pneumonia was frequent. Opie noted again the odd mix of types of pneumococci. ‘There is the notable difference that the pneumococci usually found are those types which are commonly present in the mouths of healthy men, namely, Types IV, III and atypical II and not the so-called fixed types, namely, Types I and II, which represent the usual cause of lobar pneumonia unassociated with influenza’. He hypothesized two possibilities to explain this phenomenon—either the influenza virus allowed usually non-pathogenic pneumococci to cause disease, or the exposure of recruits not previously infected led to the outcome [49].

Researchers considering the rates at multiple military camps found that it was indeed ‘southerness’ that caused greater mortality, and not race per se. They argued that two factors determined this difference. First, it ‘is an opinion generally held by medical officers in southern camps that hookworm disease and chronic malarial infection increase susceptibility to the acute respiratory diseases.’ [10] Hookworm, malaria and also niacin deficiency (pellagra) were all common in the south, and contributed to the ill health of blacks and whites [46, 50, 51]. In addition to southerners, men from rural areas were more susceptible to pneumonia than those from urban areas. They wrote, ‘The man who has lived in a densely populated area is more resistant because he has been exposed to the same bacteria before, probably many times, and has acquired more or less immunity or an increased resistance. For a converse reason, the man who has lived in a sparsely settled community is the more susceptible because he has never before … harbored these bacteria.’ [10] They compared such men with the American Indians who had been decimated by smallpox and measles brought by the Spanish after 1492.

These researchers were using the concept of ‘virgin soil’ to explain the spike in mortality. This is a complex theory about differential immunity which encompasses both evolved genetic differences and childhood exposure. Historians have challenged the validity of such arguments as applied to Native Americans, because they ignore the great cruelty and societal destruction that the invading Spanish caused, factors that led to malnutrition, social disruption and great diminution of bodily health [52]. Here I suggest that in 1918 rural peoples of any race might have had less exposure to pneumococcus in childhood and thus were more likely to develop severe symptoms as adults. There is no suggested genetic component here, and this does not rule out the importance of poverty in all its features—malnutrition, concomitant diseases, poor housing and lack of medical care. But this case study does raise as a hypothesis the idea that rural origin might well be correlated with death or severe morbidity from the influenza/pneumonia complex.

Opie was clearly familiar with prior work on pneumonia. He knew that it rarely occurred in epidemics, except in situations of crowded housing, such as military barracks, prisons, asylums or residential schools, as noted in the most prominent contemporary textbook [53]. His interest in the black troop susceptibility to the disease may well have been primed by knowledge of two major pneumonia epidemics of the early twentieth century—an outbreak among Caribbean laborers working on the Panama Canal, and a second among South African gold and diamond miners. Hundreds of thousands of black Africans were conscripted to work in miserable conditions of crowded housing, cramped mines and inadequate food. Pneumococcus was the pathogen. And from these studies Opie learned to look for social determinants of health that included diet, crowding and the mixture of isolated rural men lacking prior exposure to organisms [54].

William Crawford Gorgas had controlled the Panama epidemic by moving workers out of crowded barracks and into small huts for the workers and their families. In South Africa the company took a different course, bringing in prominent British immunologist and bacteriologist Almroth Wright in 1910 in hopes that he could create a vaccine against the pneumococcus. He first attempted to create immunity by injecting killed pneumococci, but the vaccine failed to show much efficacy. He left the field to his assistant, Spencer Lister, and returned to England. Lister was one of the first to discover that the pneumococcus existed in multiple strains or types, and that immunity to one did not cause immunity to all. He found that three types (A, B and C in his lexicon) caused 70% of the cases. So he created a new vaccine with higher numbers of killed bacilli that were type-specific. Lister demonstrated substantial decrease in incidence and mortality. But at the same time the mine owners were following the Gorgas plan, and improving the diet and living quarters of the miners. This dual program confused the relevant contributions of each intervention [54, 55].

**Figure 3.**
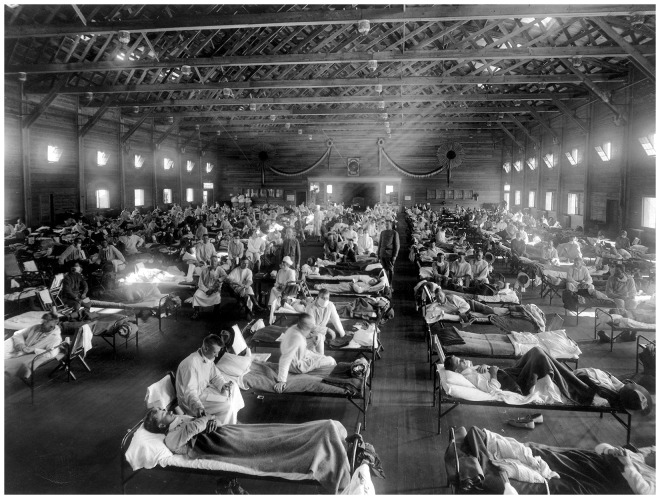
Emergency hospital during the 1918 influenza epidemic, Camp Funston, Kansas” (NCP 1603). OHA 250: New Contributed Photographs Collection, Otis Historical Archives, National Museum of Health and Medicine

Opie was committed to the ‘environmental solution’ for both influenza and pneumonia. He was convinced that ‘Influenza is a self-limited disease which, in the absence of complications implicating the lower respiratory tract, is of relatively mild character. When death occurs as the result of influenza it is with very rare, if any, exceptions, referable to pneumonia’. Accordingly, ‘The greatest source of danger to one with influenza is contact with patients who have acquired pneumonia’. The worst setting for an influenza patient was the crowded hospital ward typical of military camps overrun with flu cases. If he did not have pneumonia when brought in, he would acquire it from the next bed. Only when such pneumonias were recognized as noscomial, and ‘as much as puerperal fever or the hospital gangrene of former years’ would the situation improve [49] [Fig. 3].

Opie’s preventive measures were all about avoiding overcrowding. Overcrowding of barracks and overcrowding of troop trains had led to the deaths of ‘thousands of recruits within one month of their entrance into military service’. Similarly, the ‘overcrowding and confusion of hospital facilities in the presence of an epidemic disease’ could be alleviated with proper planning. The key step was ‘isolation of each patient with pneumonia’ as ‘the most effective way of protecting him from infection and preventing him from becoming a possible source of danger to others’. Something more was needed than the hanging of sheets between crowded beds, or placing the men so their heads and feet alternated between beds. During the American Civil War cases of smallpox or hospital gangrene were isolated in separate tents from the main hospital wards. What Opie is calling for here is the recognition that pneumonia following influenza was just as deadly, and just as contagious, as these more traditional camp hospital pathogens [49, 56].

## HISTORY OF THE PANDEMIC (II)

In late August and September of 1918 the highly mortal second wave emerged in Europe, America and Africa, and moved from military camps into the general population. As the only reconstructed influenza genomes come from after this time, there is no way at present to determine what features of the virus changed between March and September. Certainly the environment for the emergence of virulence persisted, but viral mutation (probably) set the stage for greater spread of the virus from its previous settings. The death rates were high enough to depress the overall life expectancy of the American population by 12 years. The harshest phase struck in the US civilian population in October and November 1918, followed by a smaller number of cases in early 1919 (the third wave) [5]. Such a rampant epidemic demanded government response, and those attempts at prevention took several forms. Were any of them effective at decreasing the spread of the epidemic?

Howard Markel and colleagues have asked just that question about ‘nonpharmaceutical interventions’ carried out by 43 U.S. cities from 8 September 1918 to 22 February 1919—the time limits of the second and third waves of epidemic influenza in those years. They asked whether city by city variation in mortality varied according to nonvaccine public health interventions. The interventions included mandatory (1) isolation of sick persons and quarantine of their contacts; (2) school closures; (3) bans on public gatherings. Markel *et al.* found that cities that acted early, and employed all three tactics, experienced a much milder outbreak than those who delayed or took half measures. Indeed some cities served as their own controls if the measures were not steadily applied; if after a growing mortality from influenza became evident, the city imposed these measures; then when the situation improved, the bans were lifted—and a second epidemic peak followed. Markel *et al.* [57] conclude, ‘Cities that were able to organize and execute a suite of classic public health interventions before the pandemic swept fully through the city appeared to have an associated mitigated epidemic experience’.

Markel *et al.* did not address that most iconic of 1918 influenza actions, the donning of gauze facemasks. Some communities, such as San Francisco, made the wearing of masks compulsory in public. The masks consisted of folded layers of gauze of varying thicknesses, tied on with strings. In 1920 the secretary and executive officer of the California State Board of Health revealed that studies from his board ‘did not show any influence of the mask on the spread of influenza in those cities where it was compulsorily applied’. He agreed that the idea of the mask stopping infectious particles seemed logical, so he and bacteriologist colleague Grace McMillan set out to create laboratory conditions to test the matter. They found that layers or density of gauze of sufficient thickness to stop bacilli also made breathing difficult. The wearer would breathe around the edges and otherwise remove the mask as soon as possible. Thinner gauze was more comfortable but of little use. They also surveyed users, and found many problems. A large number of masks were improperly made; some covered only the nose or only the mouth; many people wore masks in public as required by law but put them aside when out of view in private gatherings [58].

A different strategy sought to prevent the disease by means of inoculation against influenza. Or at least against the bacterium that many thought caused influenza. In 1889 Richard Pfeiffer, a German bacteriologist associated with Robert Koch, announced that he had found the causative agent of influenza. Dubbed *Bacillus influenzae* or *Haemophilis influenzae*, Pfeiffer’s bacillus was commonly found in influenza patients. (While Pfeiffer’s bacillus and the modern organism of *Hemophilis influenzae* (*H. flu*) probably overlap in identity, the mapping may not have been precise. Even Pfeiffer noted the finding at times of pseudo-influenza bacilli. *H. flu* is a common oral organism that can cause sinus, ear and respiratory tract infections). But he could not find an animal model in which to convincingly demonstrate Koch’s postulates. Bacteriological investigation of the 1918 epidemic left many physicians unconvinced about Pfeffer’s bacillus as the causative organism. Yes, they found it often, but not always [59]. Already by 1918 the concept of a different sort of microorganism, one small enough to pass through a porcelain filter, had been accepted for other diseases, such as yellow fever [60]. Leading American hygienist Victor Vaughan spoke for many when he reported, ‘Pfeiffer’s work was never accepted whole heartedly’, as the organism to which he gave his name was found in the throats of healthy people or and in other respiratory infections unconnected to influenza [9]. Still, the severity of the 1918 influenza emergency sparked a rush to prevention by any means at hand.

Desperate times called for desperate measures, and trials of a vaccine against Pfeiffer’s bacillus were widespread. Many different vaccines were developed, using the strategy of injecting killed microorganisms. Some targeted various strains of Pfeiffer’s bacillus; others added pneumococcus or other streptococci to the mix. No one regulated such trials. A doctor made up a few liters, distributed it to his clinician friends or tried it out in an institutional setting such as orphanage or asylum. Then he announced the results. As historian John Eyler reported, ‘Regardless of the vaccine they tested or the approach they used, most researchers who published the results of influenza vaccine trials in 1918 concluded their vaccine was effective.’ [61] Choosing a relevant control group bedeviled researchers. And they had little idea how many people had already engaged the virus in subclinical infections. Proper identification of influenza’s etiology as viral did not come until the 1930s, and the first effective vaccines followed later, although first researchers had to discover the different types and susceptibilities of the influenza virus [62].

## DISCUSSION

The Camp Funston study suggests that understanding the emergence of virulent influenza in 1918 means considering the outbreak’s mortality as the result of a two-stage infection, first by virus and second by bacterium. Susceptibility to these two organisms had different determinants, although some of course overlapped as well.

Susceptibility to the specific influenza virus of 1918 depended on whether the individual had childhood imprinting by the virus that circulated during the 1889–90 influenza outbreak. Influenza is highly contagious, but not every epidemic reaches all populations. So not only those born since 1889, but those in remote lands untouched by the prior outbreak would be particularly susceptible. How the virus changed at the end of August 1918 will probably never be known from genomic studies. Since the most visible feature appears to be movement from the military into civilian populations, perhaps the key mutation was in some factor that amplified ease of transmission and concordant contagiousness. The case mortality rate appears to have stayed about the same in most populations—5%–but many more people were sick from the virus than in a usual influenza epidemic. This may reflect actual spread, or the fact that more people not only acquired the virus, but also became actively sick with it. In any epidemic there are silent, mild cases.

Susceptibility to the bacteria that caused the following pneumonia, particularly pneumococcus, had different determinants. Bacterial pneumonias rarely occur in epidemics; the best documented of such outbreaks involved the congregation of soldiers or workers in crowded barracks. This was true when ex-slaves assembled in St. Louis for induction into the Union army in 1863, as it did when early twentieth century black workers were brought to the Panama Canal worksite or the mines of South Africa [52, 63]. Such situations support World War I observations that rural men with minimal prior exposure to pneumococcus, particularly when gathered into crowded environments where spittle and hands easily spread bacteria, combined to cause pneumonia outbreaks. Pneumonia might have occurred without the precedent influenza epidemic, as it did in the situations listed above. That the influenza virus acted directly to counter bronchial immune defenses amplified bacterial reproduction which in turn spread both microorganisms.

It was only in 1920 that the United States Census demonstrated, for the first time, that more Americans lived in municipalities of 2500 people or more, than lived in rural areas [64]. So roughly half of the troops who served in the US army in World War I came from rural areas, and many had previously never left their home counties. ‘Ruralness’ could determine likelihood of exposure to the 1889 influenza as well as certain strains of pneumococcus. Southern ‘ruralness’ made prior malnutrition and co-infection with malaria and hookworm likely. The war brought all these men together with city youth in neo-urban encampments of high population density. In general, the war moved people from the immune environment of their home dwellings into contact with immune foreigners of all sorts. The importance of ‘ruralness’ as a hypothesis is presented here from one case study; analysis of other locales is needed for broader generalization.

Wartime conditions created prime environments for the emergence of virulence. New recruits and men at the front were all jammed together in crowded conditions, allowing for rapid movement of virus from the sick to the well. In the military the ill influenza patient would have been transported by others to the hospital. He did not even need to be ambulatory in order to carry the virus to others. And his caregivers obligingly transported him into the influenza/pneumonia ward, where he could catch a secondary bacterial infection if he had not yet acquired it [28].

World War 1 transformed environments globally. Men moved into areas unfamiliar to their immune systems, and in turn their microflora transformed the environments into which they entered. In World War I the seasoning had to happen over and over again as men traveled through the various spaces of the massive conflict. In the Civil War Robert E Lee evolved a strategy of letting new recruits spend time in an induction camp so they could get over having measles and such before they moved into regular military life [56]. Opie called for a similar seasoning, but there was no time for it in the hurried ramping up of the American military response to World War I [49].

Modern vaccinations limit the exposure of the developed world to influenza and pneumonia, although their power has limitations. But it takes 6–9 months to develop influenza vaccines against novel strains; the 2009 H1N1 epidemic had already moved through the summer camps and elementary schools in Durham County, North Carolina by the time the new vaccine appeared [65]. The influenza virus most prominent in 2017–18 evaded the available vaccine in multiple cases, as was widely reported in the media [66, 67]. The modern pneumococcus vaccines cover multiple strains, but are least effective in the elderly, who are most at risk from the organism. Only 77% were protected in one nursing home study, for example [68].

More to the point is that the pneumonias that killed influenza patients in 1918 can now be treated by antibiotics. If, that is, the person has access to appropriate medications. This caveat may exclude those in the developed world who lack health insurance or geographic proximity to modern hospital care. In resource poor countries the problem could be more extreme. Even in the most developed countries there are limitations in the availability of ventilators; such assistance may be key in rapidly moving pneumonias while awaiting antibiotic response. There is no reason for complacency.

## Funding

Funding provided by an Arts and Sciences Research Grant, Duke University.

**Conflict of interest**: None declared.
